# Short-Term Neonatal Outcomes Following Early-Term vs. Full-Term and Late-Preterm Births: Insights from a Retrospective Cohort Study

**DOI:** 10.3390/children12121693

**Published:** 2025-12-15

**Authors:** Nadav Kadosh, Ron Zeitouni, Roni Shlesinger Schwartz, Smadar Eventov-Friedman, Noa Ofek Shlomai

**Affiliations:** 1 Department of Pediatrics, Hadassah Medical Center, Faculty of Medicine, Hebrew University of Jerusalem, Jerusalem 91120, Israel; 2 Department of Military Medicine and “Tzameret”, Hebrew University of Jerusalem and Medical Corps, Jerusalem 91120, Israel; 3 Department of Neonatology, Hadassah Medical Center, Faculty of Medicine, Hebrew University of Jerusalem, Jerusalem 91120, Israelsmadaref@hadassah.org.il (S.E.-F.)

**Keywords:** full term, early term, late preterm, elective caesarean section, short term outcome, breast feeding

## Abstract

**Highlights:**

**What are the main findings?**

• Early term birth is associated with increased short-term morbidity, and potentially with long-term adverse outcomes.
• Birth at early term has been linked to greater weight loss, lower breastfeeding rates, higher respiratory morbidity, and prolonged hospital stays.

**What are the implications of the main findings?**

• In treating early term infants’ neonatologists should bear in mind the vulnerabilities of this group of infants.
• Elective caesarean delivery at early term should be limited to carefully selected clinical indications, and should not be adopted as routine practice.

**Abstract:**

Background: Conceptualizing gestation as a developmental continuum highlights that even among term infants, distinct subgroups—such as early-term and full-term infants—may exhibit meaningful differences in morbidity and clinical outcomes. Objective: To compare early neonatal outcomes among late-preterm (LP), early-term (ET), and full-term (FT) infants. Methods: A retrospective observational study analyzed data of infants born between 34^+0^ and 41^+6^ weeks at Hadassah Medical Centers in 2023. Infants were stratified by gestational age. Late-preterm was defined as birth between 34 − 36 + 6 weeks of gestation, early-term 37 − 38 + 6 weeks, and term birth between 39 − 41 + 6 weeks. Primary outcome was length of stay (LOS), secondary outcomes included NICU admissions, respiratory support, feeding type, weight loss, and re-hospitalizations within the first year following discharge. Results: ET infants had intermediate outcomes between LP and FT groups. LOS and respiratory support needs were higher in ET than FT infants. NICU admissions were significantly more frequent in LP infants; ET infants exhibited higher weight loss and more frequent elective cesarean deliveries compared to FT infants. Readmission rates were higher in ET compared to FT infants. Conclusions: ET infants, while more stable than LP infants, experienced increased short-term morbidity compared to FT neonates. These findings support minimizing elective delivery before 39 weeks unless clinically indicated.

## 1. Introduction

Term birth is defined as delivery between 37 weeks and 41 weeks and 6 days of gestation [1], whereas the term late-preterm infants refers to infants born between 34 and 36 + 6 weeks [2]. Given our understanding that gestation is a continuum [3], it is unreasonable to unify the 6 weeks considered term delivery as one gestational group in terms of expected requirements and outcomes [4]. We have therefore defined two subgroups within this continuum, early-term (ET) infants, born from 37 + 0 to 38 + 6 weeks of gestation, and full-term (FT) infants, born beyond 39 + 0 weeks of gestation. This aligns with the latest updates of the American Academy of Pediatrics regarding at-risk neonatal populations [5]. Late-preterm infants account for 7–9% of all births and 70–80% of all preterm births [6]. LP infants have been widely studied and were found to exhibit higher rates of respiratory distress, hypoglycemia, feeding difficulties, and neurodevelopmental challenges compared to full-term infants [2,3,6,7]. LP infants have been found to have increased short- and long-term morbidity when compared to FT infants. Short-term morbidity includes hypothermia, hyperbilirubinemia, feeding difficulties, and respiratory distress. In addition, an increased rate of pulmonary disorders through childhood and adolescence, learning difficulties, and minor cognitive deficits are found in both late-preterm and early-term infants [5,8]. Respiratory-related hospitalizations up to 18 years of age were associated with early-term birth in a large cohort of over 200,000 infants [9]. Obstructive sleep apnea was also increased in early-term infants, inversely related to gestational age in the same cohort [10]. Early-term birth was also independently associated with an increased incidence of long-term cardiovascular and hematological morbidity and hospitalizations [11,12]. In addition, early-term infants were more likely to have endocrine and metabolic disorders, specifically obesity and type I diabetes, than full-term and post-term infants [13]. Despite increasing recognition of the clinical significance of early-term births, the medical literature remains relatively sparse in studies that comprehensively evaluate neonatal outcomes in this population. In a study of over 13,000 women who have undergone an elective cesarean section (CS), Tita et al. found that delivery prior to 39 weeks of gestation was significantly associated with neonatal short-term adverse outcome, including respiratory morbidity and admission to the neonatal intensive care unit (NICU). Moreover, these were higher in infants born at 37 weeks of gestation when compared to infants born at 38 weeks [14]. These results were also demonstrated in studies investigating transient tachypnea of the newborn (TTN) and NICU admissions in infants born via elective CS before and after 39 weeks in both resource-rich and resource-limited countries [15,16,17,18]. Compromised outcome for infants born at 37 weeks was demonstrated in all countries who participated in a WHO survey in 2014 [19]. A systematic review of 29 studies demonstrated a decline in respiratory morbidity, jaundice, hypoglycemic episodes, and mortality between infants born at 37 and beyond 39 weeks of gestation [20]. Glavind et al. showed that women having an elective CS at 39 weeks have an increased risk of labor-induced CS, compared to 38 weeks; however, maternal morbidity was comparable between the groups [17]. In a study conducted over 11 years and including around 50,000 infants, NICU admissions were more common in early-term infants when compared to full-term infants. The main diagnoses were TTN and hypoglycemia [21]. Furthermore, in a study of over 755,000 live singleton births, the authors found that birth at 37 weeks of gestation significantly increases both neonatal morbidity and intrapartum mortality compared to infants born at 39–41 weeks [22].

The American Academy of Pediatrics has defined late-preterm and early-term infants as at-risk populations, with increased morbidity, mortality, and prolonged hospital stay and health care costs [5]. However, rates of early-term births remain high across the globe, with a substantial percentage of these infants born via elective CS [23,24].

The aim of this study was to evaluate early neonatal outcomes of early-term (ET) infants compared to late-preterm (LP) term and full-term (FT) infants.

## 2. Methods

### 2.1. Study Design and Patient Population

This retrospective observational study utilized data extracted from the institutional electronic medical records of Hadassah Medical Centers (Ein Kerem and Mount Scopus hospitals). Both hospitals have a level III NICU. We analyzed records of 660 infants born between 34 + 0 and 41 + 6 weeks of gestation from March to June 2024. Patients were selected by consecutive birth order according to hospitals delivery records. Due to the retrospective nature of the study, informed consent was waived as the study was retrospective, and the data were de-identified prior to analysis.

The primary outcome was length of stay (LOS), measured in days. Secondary outcomes included neonatal characteristics (mode of delivery—emergency cesarean section (CS), pre-planned/elective CS, or vaginal birth), antenatal corticosteroids, birth weight, weight loss > 8% during initial hospitalization [25]), and clinical course (NICU stay and duration, breast feeding rates, respiratory support in delivery room, respiratory diagnoses, hypoglycemic episodes, hyperbilirubinemia requiring phototherapy, and hypothermia < 36.5 °C on admission). Lactation consultants were available throughout hospital stay to all mothers at least once, and/or if the nursing staff identified difficulties in breastfeeding in the nurseries, while in the NIC’s, the consultations are provided daily during the first days after delivery. In addition, rates of re-hospitalization within the first year of life were evaluated.

Exclusion criteria included congenital anomalies requiring major surgery during initial hospitalization and neonatal death within the first 96 h of life.

Infants were stratified into three groups according to gestational age: late-preterm (LP), 34 + 0 to 36 + 6 gestational age (GA), early-term (ET), (37 + 0 to 38 + 6 GA, and full-term (FT), 39 + 0 to 41 + 6 GA.

This study was approved by the Hadassah ethical board committee, approval number HMO-0019-24, and was performed in accordance with the Helsinki declaration.

### 2.2. Statistical Analysis

Sample size calculations were based on expected differences in length of stay (LOS) among early-term, late-preterm, and full-term groups. A total of 220 newborns per group were included to ensure 90% power with a 5% significance level. Variables with low incidence, such as advanced respiratory support, TPN, and surfactant administration, were excluded from the final analysis. Categorical variables were analyzed using chi-square or Fisher’s exact test and continuous variables with Pearson correlation coefficient. Overall comparisons of continuous variables across the three study groups were conducted using a one-way Analysis of Variance (ANOVA) test, with Levene’s test for the equality of variances and the Brown–Forsythe robust test of equality of means when unequal variances were found. The Dunnett T3 test was used for multiple pairwise comparisons, with adjustment of the significance level. Variables which were significantly associated with dichotomous outcomes in the univariate approach were entered into the multiple logistic regression model. This model was applied using the stepwise, forward, likelihood ratio method, ensuring that multicollinearity does not affect the variables entered into the model.

## 3. Results

A total of 660 newborns were included in the study, evenly divided (220 infants in each group) into three groups based on gestational age group. Mean gestational age was 38.5 weeks, and mean birth weight was 3002.4 g. Of the 660 mothers, 164 (24.8%) were primiparas, and 10 mothers (1.5%) received at least one course of corticosteroids before delivery. Baseline characteristics of the 660 neonates are depicted in Table 1.

Comparison of the three groups revealed that ET infants had a shorter mean hospital stay than LP infants, although longer than FT infants. Mean birth weight was the lowest in LP infants, increasing with gestational age as expected. Weight loss of over 8% during hospitalization was observed more frequently in ET neonates when compared to FT neonates but less frequently than in LP neonates.

Pairwise comparisons between ET infants, LP, and FT infants are depicted in Table 2 and Table 3. Mode of delivery differed between the groups, with more LP neonates born via emergency CS, more ET neonates born via elective CS, and more FT neonates born via vaginal delivery. Significant differences were observed between groups in both overall comparison and pairwise comparisons (*p* < 0.001). Respiratory support requirements in the delivery room (positive pressure ventilation using mask or intubation) were highest in LP neonates, followed by ET neonates, with FT neonates required the least intervention. Regarding feeding type, more ET and FT neonates were fed exclusively by breastmilk at discharge compared to LP infants (*p* < 0.001).

**Figure 1:**
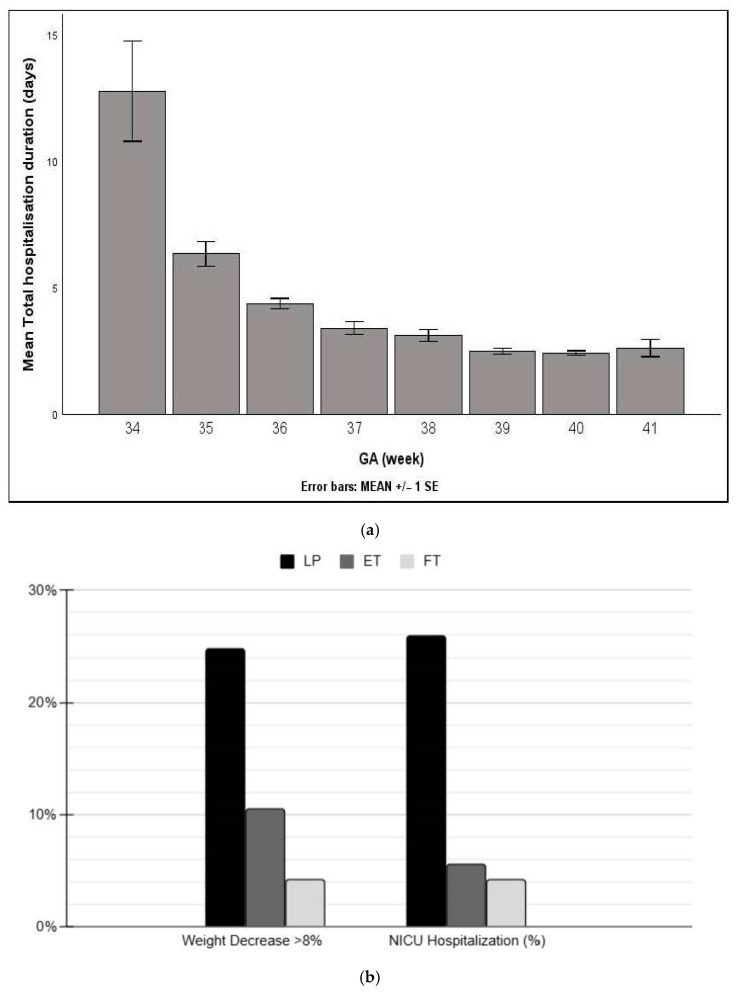
(**a**): Length of stay according to gestational age; (**b**): weight loss and NICU admission for late-preterm, early-, and full-term infants.

NICU admissions occurred in 78 neonates (11.8%), with LP neonates showing significantly higher admission rates and longer stays compared to ET and FT neonates (*p* < 0.001). No significant differences in NICU outcomes were observed between ET and FT groups (*p* = 0.502). LP neonates demonstrated significantly higher rates of hypothermia, hypoglycemia, jaundice, and respiratory distress compared to ET neonates (*p* < 0.001 for all outcomes). ET and FT neonates showed no significant differences in these clinical variables. FT neonates had the highest incidence of Meconium Aspiration Syndrome (MAS) compared to LP and ET groups. Figure 1a illustrates the rates of >8% weight loss and NICU admissions. Figure 1b illustrates length of stay according to gestational age.

**Figure 2:**
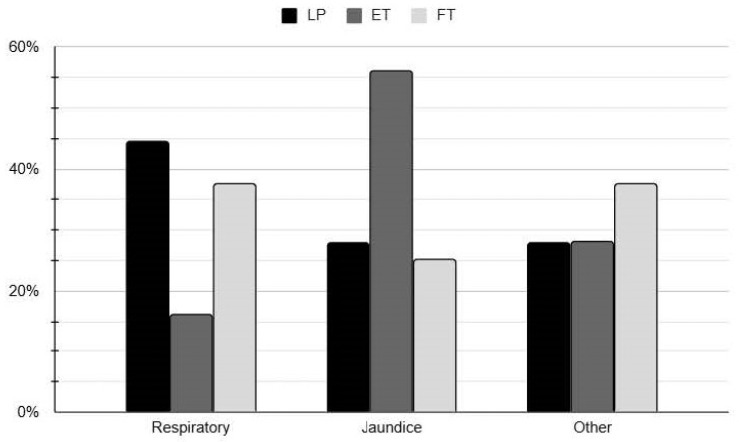
Re-hospitalization causes in late-preterm, early-, and full-term infants.

Re-hospitalization within the first year of life was more frequent in ET neonates compared to FT neonates (*p* = 0.005), though no significant difference was found in the specific diagnoses leading to hospitalization. While the overall re-hospitalization rate did not significantly differ between LP and ET neonates (*p* = 0.033), LP neonates were more frequently hospitalized due to respiratory-related causes. Figure 2 illustrates the reason for readmission in LP, ET, and FT infants in our cohort. ET neonates trended towards an increased rate of hospitalization due to hyperbilirubinemia compared to LP and FT neonates, though this finding did not reach statistical significance. This may be due to the small numbers of readmitted infants.

Logistic regression identified birth weight (*p* < 0.001) and gestational age group (*p* = 0.002) as independent predictors of NICU admission, with gestational age providing additional explanatory value beyond birth weight.

## 4. Discussion

In this study, we aimed to evaluate and compare early neonatal outcomes across three gestational age groups: late-preterm, early-term, and full-term infants. Our findings revealed significant differences in early outcomes among the gestational age groups. ET infants experienced prolonged hospital stays, greater weight loss, and an increased need for respiratory support compared to FT infants. Late-preterm infants demonstrated a higher incidence of these outcomes compared to early-term infants. Overall, the LP infant group was found to have more adverse outcomes compared to ET and FT infants. These outcomes include NICU admission rate, requirement of respiratory support and phototherapy, longer length of stay, hypothermia, hypoglycemia, and more. Given the extensive literature addressing late-preterm infants, our discussion primarily centers on the early-term group to highlight their distinct clinical outcomes and implications.

Our data revealed increased rates of elective CS in ET infants when compared to both LP and FT infants. Globally, cesarean section rates have risen significantly over the past two decades [26], with a particularly notable increase in rates for early-term deliveries. This trend may have important implications for short-term neonatal outcomes, especially in relation to respiratory morbidity. At Hadassah hospitals, discharge criteria for neonates include stable respiratory function, keeping body temperature within normal range, successful oral feeding, and adequate weight gain. Length of hospital stay was significantly increased in ET infants compared to FT infants within our cohort. This finding is consistent with the current literature [27,28]. Notably, prolonged hospitalization has been associated with lower breastfeeding rates, increased parental anxiety, disrupted mother–infant bonding, and elevated health care costs.

Our data did not demonstrate a statistically significant higher rate of NICU admissions in ET infants compared to FT infants. However, increased NICU admissions in ET infants was demonstrated in several recent studies [16,18,20,27,28]. Furthermore, NICU admissions were reduced following implementation of a policy to eliminate elective ET cesarean sections [21]. At our hospitals, conditions such as hypoglycemia, hypothermia, hyperbilirubinemia, and mild respiratory distress—common reasons for admission of ET infants to the NICU—are frequently managed in the well-baby nursery under appropriate monitoring and intensified care. This may explain the observed discrepancy.

Pathological weight loss exceeding 8% was more prevalent among ET infants compared to their FT counterparts. Several studies have reported that excessive weight loss and breastfeeding difficulties are inversely proportional to gestational age and are more common following cesarean deliveries, influencing delayed onset of lactation [29,30]. In our cohort, breastfeeding rates among ET infants were higher than those observed in LP infants but lower than in FT infants. Overall, breastfeeding rates remained relatively low compared to WHO recommendations for exclusive breastfeeding during the first 6 months of life. In our medical centers, breastfeeding rates are substantially lower, ranging from 20 to 70%. Increasing breastfeeding rates has been recognized as a national health priority actively promoted by the Ministry of Health [31]. Additional studies have similarly demonstrated that ET infants are at higher risk for delayed breastfeeding initiation, lower rates of exclusive breastfeeding, and shorter breastfeeding duration overall [32,33]. We observed a higher requirement for respiratory support among ET infants compared to FT infants; however, our data did not demonstrate statistically significant differences in specific respiratory diagnoses. This finding is consistent with previous studies that have reported not only increased needs for respiratory support but also higher incidence of respiratory distress syndrome and transient tachypnea of ET infants relative to FT infants [34,35]. Furthermore, ET birth has been associated with increased rates of neonatal readmission due to lower respiratory tract infections, including pneumonia and bronchiolitis [36]. Some studies have suggested a role for antenatal steroids in planned ET deliveries, aimed at reducing respiratory morbidity in this population [37,38].

In our cohort, hospital readmission rates during the first year of life were higher in ET compared to FT infants. While late-preterm infants were primarily re-hospitalized due to respiratory illnesses, the predominant cause of readmission among ET infants was hyperbilirubinemia. This pattern is consistent with findings from a large study encompassing over 25,000 infants, which also reported increased rates of readmission due to hyperbilirubinemia among ET infants [28]. Studies examining hospital admissions up to 18 years of age have revealed increased hospitalizations due to respiratory, endocrine, cardiological, and hematological morbidities in infants born in early term compared to term and post-term infants [9,10,11,12,13].

Evidence regarding long-term developmental differences between ET and FT infants is accumulating in the literature [31,39] but is unfortunately beyond the scope of this study.

This study has several strengths, including the relatively large cohort of infants born at a single center with standardized management policies. Another strength is the specific focus on early-term infants compared with their full-term counterparts. The main limitation of this study is its retrospective design, which inherently relies on medical records and is therefore subject to the accuracy and completeness of data collection.

In conclusion, accumulating evidence indicates that ET birth is associated with increased short-term morbidity and potentially with long-term adverse outcomes. Birth at early term has been linked to greater weight loss, lower breastfeeding rates, higher respiratory morbidity, and prolonged hospital stays. Elective cesarean delivery at early term should therefore be limited to carefully selected clinical indications and should not be adopted as routine practice.

## Figures and Tables

**Table 1:** Population characteristics.

Variable	
**Gestational Age (weeks ± SD)**	38.05 ± 1.93
**Birth Weight (g ± SD)**	3002.46 ± 557.75
**First Delivery n(%)**	164 (24.8%)
**Antenatal Corticosteroids n(%)**	10 (1.5%)
**Mode of Delivery n(%)**	
Normal Vaginal Delivery (NVD)	417 (63.2%)
Emergency C-Section	113 (17.1%)
Elective C-Section	84 (12.7%)
Instrumental	46 (7.0%)

**Table 2:** Pairwise comparisons, late-preterm vs. early-term.

Variable N (%)	Late-Preterm n = 220	Early-Term n = 220	*p*-Value	OR (CI 95%)
**Length** **of Stay, days (m)**	6.23 (median = 4.00)	3.25 (median = 3.00)	<0.001	(1.91–4.04)
**Birth Weight, g (m)**	2539.54	3046.20	<0.001	(−605–407)
**NICU Hospitalization**	57 (25.9%)	12 (5.5%)	<0.001	6.0 (11.6–3.15)
**Weight Decrease >8%**	54 (24.7%)	23 (10.5%)	<0.001	2.79 (4.73–1.64)
**Mode of Delivery**			<0.001	
NVD ^1^	102 (46.4%)	131 (59.5%)		0.58 (0.83–0.41)
Emergency CS	80 (36.4%)	22 (10.0%)		5.14 (8.75–3.02)
Elective CS	21 (9.5%)	57 (25.9%)		0.3 (0.5–0.1)
Instrumental	17 (7.7%)	10 (4.5%)		1.78 (3.87–0.8)
**Respiratory Support**	59 (26.8%)	29 (13.2%)	0.001	2.41 (3.95–1.48)
**Any Respiratory Disease**	29 (13.2%)	7 (3.2%)	<0.001	4.62 (10.8–1.98)
TTN ^2^	21 (9.5%)	6 (2.7%)	0.003	3.76 (9.52–1.49)
**Type of Feeding**			<0.001	
Full Breastfeeding	18 (8.2%)	48 (21.8%)		0.32 (0.57–0.18)
Formula	202 (91.8%)	172 (78.2%)		3.13 (5.58–1.76)
**Hypothermia at Admission**	55 (25.0%)	23 (10.5%)	<0.001	2.86 (4.84–1.68)
**Hypoglycemia**	91 (41.4%)	27 (12.3%)	<0.001	5.04 (8.18–3.11)
**Phototherapy**	71 (32.3%)	29 (13.2%)	<0.001	3.14 (5.08–1.94)
**Re-hospitalization after Discharge**	36 (16.4%)	25 (11.4%)	0.033	1.53 (2.64–0.88)
Respiratory	16 (44.4%)	4 (16.0%)	0.02	4.2 (14.7–1.2)
Hyperbilirubinemia	10 (27.8%)	14 (56.0%)	0.026	0.3 (0.89–0.1)
**Antenatal Corticosteroids**	9 (4.1%)	1 (0.5%)	0.01	9.34 (74–1.18)

^1^ Normal vaginal delivery, ^2^ transient tachypnea of the newborn.

**Table 3:** Pairwise comparisons, early-term vs. full-term.

Variable	Early-Term n = 220	Full-Term n = 220	*p*-Value	OR (95% CI)
**Length** **of Stay, days (m)**	3.25 (median = 3.00)	2.51 (median = 2.00)	<0.001	
**Birth Weight g (m)**	3046.20	3421.65	<0.001	2.73 (6.04–1.23)
**NICU Hospitalization**	12 (5.5%)	9 (4.1%)	0.502	1.35 (0.56–3.27)
**Weight Decrease >8%**	23 (10.5%)	9 (4.1%)	0.011	2.73 (1.23–6.04)
**Mode of Delivery**			<0.001	0.15 (0.09–0.27)
NVD ^1^	131 (59.5%)	184 (83.6%)		0.53 (0.271–0.09)
Emergency CS	22 (10.0%)	11 (5.0%)		2 (4.55–0.88)
Elective CS	57 (25.9%)	6 (2.7%)		21.4 (52.8–8.7)
Instrumental	10 (4.5%)	19 (8.6%)		0.55 (1.21–0.23)
**Respiratory Support**	29 (13.2%)	15 (6.8%)	0.024	2.08 (1.08–3.99)
**Any Respiratory Disease**	7 (3.2%)	9 (4.1%)	0.610	
TTN ^2^	6 (2.7%)	3 (1.4%)	0.503	2.68 (11.3–0.63)
**Type of Feeding**			0.08	1.47 (0.95–2.27)
Full Breastfeeding	48 (21.8%)	64 (29.1%)		
Formula	172 (78.2%)	156 (70.9%)		
**Hypothermia at Admission**	23 (10.5%)	18 (8.2%)	0.412	1.33 (2.56–0.69)
**Hypoglycemic episodes**	27 (12.3%)	14 (6.4%)	0.033	2.06 (1.05–4.05)
**Phototherapy**	29 (13.2%)	28 (12.7%)	0.887	1.04 (1.88–0.58)
**Re-hospitalization after Discharge**	25 (11.4%)	8 (3.6%)	0.005	3.39 (1.49–7.68)
Respiratory	4 (16.0%)	3 (37.5%)	0.32	0.32 (0.05–1.89)
Jaundice	14 (56.0%)	2 (25.0%)	0.225	3.83 (0.64–22.8)
**Antenatal Corticosteroids**	1 (0.5%)	0 (0%)	1.000	

^1^ Normal vaginal delivery, ^2^ transient tachypnea of the newborn.

## Data Availability

Data will be made available upon request.
